# Evaluation of Right Ventricular Myocardial Properties Using Systolic Myocardial T1 Mapping

**DOI:** 10.7759/cureus.67797

**Published:** 2024-08-26

**Authors:** Yuki Sasaki, Hideharu Oka, Kouichi Nakau, Yuki Shibagaki, Keita Ito, Rina Imanishi, Sorachi Shimada, Yuki Akiho, Kazunori Fukao, Sadahiro Nakagawa, Kunihiro Iwata, Satoru Takahashi

**Affiliations:** 1 Pediatrics, Asahikawa Medical University, Asahikawa, JPN; 2 Pediatric Cardiology, Asahikawa Medical University, Asahikawa, JPN; 3 Radiology, Asahikawa Medical University Hospital, Asahikawa, JPN

**Keywords:** right ventricular myocardium, systolic phase, cardiac magnetic resonance (cmr), t1 mapping, congenital heart disease (chd)

## Abstract

Introduction

Myocardial properties can be quantitatively evaluated using myocardial native T1 values (nT1) obtained using cardiac magnetic resonance imaging (CMR). In terms of myocardial wall thickness, the left ventricular nT1 is easy to measure, but the right ventricular nT1 is difficult. Patients with congenital heart disease often develop right ventricular overload. If right ventricular nT1 can be measured consistently, inflammation and fibrosis of the right ventricular myocardium can be quantitatively evaluated. We aimed to determine whether T1 mapping during systole can be used to evaluate right ventricular myocardial properties.

Methods

T1 mapping was performed at diastole and systole. Systolic T1 mapping was calculated from diastolic T1 mapping and cine images. The myocardial properties of both ventricles were evaluated in 13 healthy volunteers (21-26 years old) and 12 patients with right ventricular overload (12-41 years old) who underwent CMR examination at our hospital.

Results

From the analysis of left ventricular nT1, we found that nT1 did not change significantly during the cardiac cycle. However, right ventricular nT1 changed between diastole and systole because the right ventricle is affected by blood. Although there was no difference in right ventricular diastolic nT1 between the patients and volunteers (1,346.8 vs. 1,347.6 ms, p = 0.852), the right ventricular systolic nT1 was significantly higher in patients than in volunteers (1,312.7 vs. 1,233.8 ms, p = 0.002). This indicates that right ventricular myocardial damage occurs in patients with right ventricular overload.

Conclusion

Systolic right ventricular myocardial T1 mapping allows assessment of right ventricular myocardial properties. The right ventricular myocardial systolic nT1 is useful for evaluating myocardial damage due to right ventricular stress and myocardial injury. Measuring right ventricular nT1 may allow consideration of early therapeutic intervention.

## Introduction

The prognosis of patients with congenital heart disease (CHD) has improved thanks to advances in medical technology [1]. The number of patients surviving CHD in Japan continues to rise by approximately 10,000 annually, leading to an increasing number of CHD patients reaching adulthood [2]. Various problems occur in the long term after surgery for CHD, and right heart failure is one of the pathologies that should be noted. For example, the tetralogy of Fallot is a problem of pressure overload and volume overload due to postoperative pulmonary valve stenosis and regurgitation. Right heart failure is determined by dilation of the right ventricle and decreased contractile force; if it worsens, reoperation is required [3]. Reaching the criteria for reoperation may take years, and progress is often observed during this time. However, during this period, the right ventricular myocardium gets damaged, and it is difficult to evaluate the degree of damage to the myocardium.

In recent years, T1 mapping has become popular in cardiac magnetic resonance imaging (CMR) examinations, enabling quantitative evaluation of myocardial properties [4]. Although evaluation of myocardial fibrosis using delayed contrast imaging was standard, it included problems such as the inability to detect diffuse lesions and the need for a contrast agent [5]. T1 mapping can measure the myocardial native T1 value (nT1) without a contrast agent and evaluate diffuse lesions; therefore, an objective investigation of changes in myocardial properties is possible. nT1 is known to increase in the presence of myocardial inflammation or fibrosis and amyloidosis, whereas it decreases in the presence of iron and fat deposits and Fabry disease [6]. Care must be taken to avoid a nonspecific increase in nT1 due to blood when measuring the T1 values [7]. Since the T1 value of blood is higher than that of the myocardium, if the region of interest (ROI) is surrounded by blood components, the T1 value will increase significantly. As for the left ventricle, the thickness of the myocardial wall is sufficient; therefore, blood components are less of a problem when surrounding the ROI. On the other hand, in the right ventricle, the myocardial wall is thin and the endocardial surface is rough. Therefore, the boundary between the blood and myocardial components tends to be unclear, making it difficult to measure the right ventricular nT1 [8]. Diastolic right ventricular nT1 tends to be elevated due to blood.

If this problem can be overcome and T1 mapping can be used to quantitatively evaluate right ventricular myocardial properties, it may be useful for understanding the pathology of the right heart system. If right ventricular nT1 can be measured consistently, inflammation and fibrosis of the right ventricular myocardium can be quantitatively evaluated. In systole, the thickening of the myocardial wall may provide sufficient ROI to evaluate right ventricular nT1. Therefore, we aimed to determine whether systolic T1 mapping is a viable method for assessing right ventricular myocardial properties, particularly in patients with right ventricular overload.

## Materials and methods

**Figure 1:**
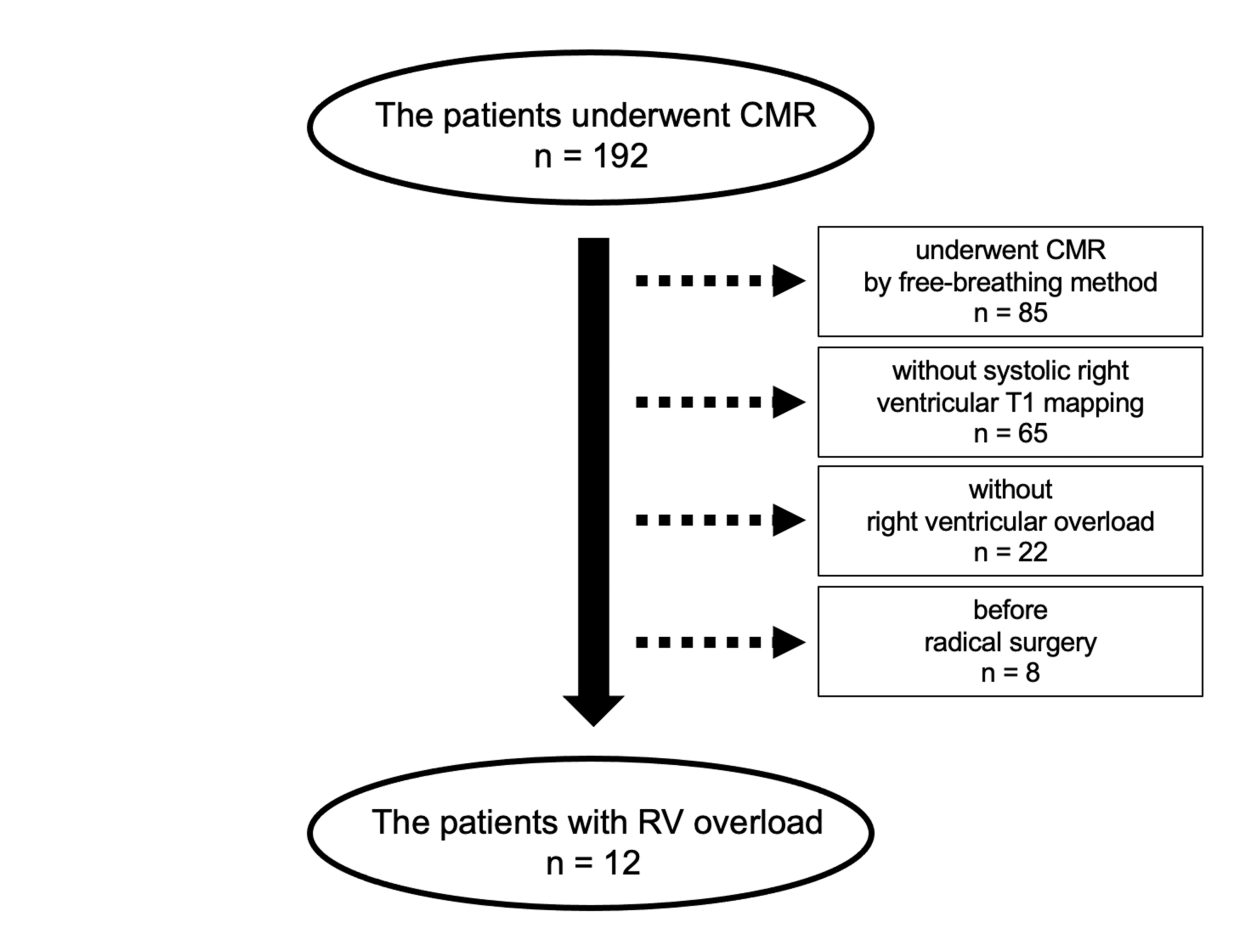
Patient flow CMR, cardiac magnetic resonance imaging; RV, right ventricular

We conducted a retrospective study that included patients who underwent CMR in the Department of Pediatrics of Asahikawa Medical University Hospital, Asahikawa, Japan, between March 2020 and December 2023. The patients underwent MRI for hemodynamic parameter evaluation as part of routine follow-up or surgical planning and were examined for diastolic and systolic T1 mapping. There were 192 patients, and we excluded 85 patients who underwent CMR by the free-breathing method, 65 patients without systolic right ventricular T1 mapping, 22 patients without right ventricular overload, and eight patients before radical surgery (Figure 1). Patients who underwent CMR by the free-breathing method were excluded due to the possibility that motion artifacts may alter T1 values.

We investigated 12 patients (12-41 years old) with right ventricular overload, and they were as follows: tetralogy of Fallot (n = 5), transposition of the great artery (n = 3), idiopathic pulmonary artery hypertension (n = 2), double outlet right ventricle (n = 1), and pulmonary atresia with intact ventricular septum (n = 1). These patients with CHD underwent postsurgical repair. Thirteen volunteers (21-26 years old) who underwent CMR examinations were included. Echocardiography was performed to exclude any cardiovascular pathology in the healthy subjects. This study was conducted in compliance with the standards of the Declaration of Helsinki and the current ethical guidelines and was approved by our institutional ethics board (approval number 21163). Written informed consent was obtained from all participants.

All cardiac imaging examinations were performed using MAGNETOM Vida (Siemens Healthcare, Erlangen, Germany) with a 3.0 Tesla MR system. The modified Look-Locker inversion recovery sequence with motion correction was used for T1 mapping. The images required for T1 mapping were taken in one-six cardiac short-axis slices (basal, mid, and apex). T1 mapping was performed under breath-holding conditions in all patients. The other scan parameters were as follows: field of view, 360 × 360 mm; slice thickness, 8 mm; flip angle, 35°; matrix size, 256 × 144; base resolution, 256; phase resolution, 144 (66%); reduce field of view, 85.2%; pixel size, 1.4 × 1.4 × 8.0 mm^3^; acceleration factor, 2; echo time, 1.06 ms; repetition time, 2.53 ms; and shot mode, true fast imaging with steady-state precession pulse sequence using the 5b(3b)3b scheme. A workstation (Cvi42, Circle, Cardiovascular Imaging, Calgary, Canada) was used for analysis.

**Figure 2:**
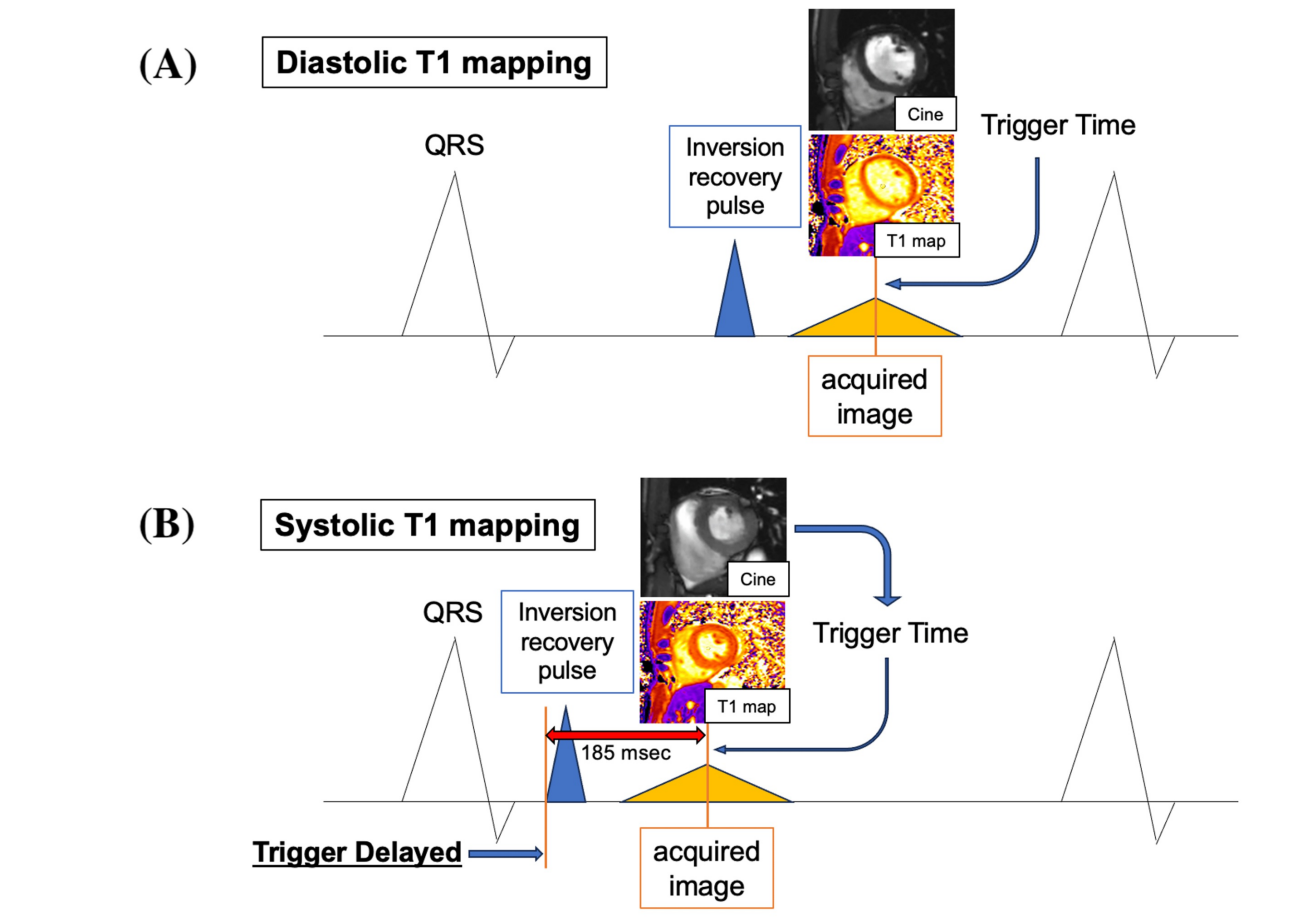
Setting of systolic T1 mapping (A) Diastolic T1 mapping. We obtained the device’s setting time (inversion recovery pulse time) and imaging time (trigger time). This time difference was found to be 185 ms. (B) Systolic T1 mapping. The setting time (trigger delayed time) for systolic T1 mapping was calculated by subtracting 185 ms from the systolic imaging time of the cine MRI.

In our study, systolic T1 mapping was performed using the following method (Figure 2).

We calculated the time difference between the setting time on the device side when capturing the diastolic T1 mapping and the capture time of the completed diastolic T1 mapping images. This difference is the time required for the T1 mapping process. By subtracting this time difference from the systolic imaging time of the cine MRI image, it became possible to determine the setting time of the systolic T1 mapping on the device side, and the systolic T1 mapping images became possible. When we obtained the setting time and imaging time on the device side of the diastolic T1 mapping of 25 participants who had been studied, the setting time on the device side was 236.2 ± 132.4 ms, the shooting time was 421.0 ± 132.6 ms, and the time difference was found to be 184.8 ± 0.84 ms. Therefore, the setting time on the device side for systolic T1 mapping was calculated by subtracting 185 ms from the systolic imaging time of cine MRI to enable the systolic T1 mapping images. We confirmed visually that the systolic T1 mapping images taken as described above were in the systolic phase.

All parameters are expressed as mean ± SD. Statistical differences were determined using the Mann-Whitney U test. Statistical significance was set at p < 0.05. Statistical calculations were performed using IBM SPSS Statistics for Windows, Version 28.0 (Released 2021; IBM Corp., Armonk, NY, USA). This article was previously posted to the Research Square preprint server on January 17, 2024.

## Results

LV T1 mapping

**Figure 3:**
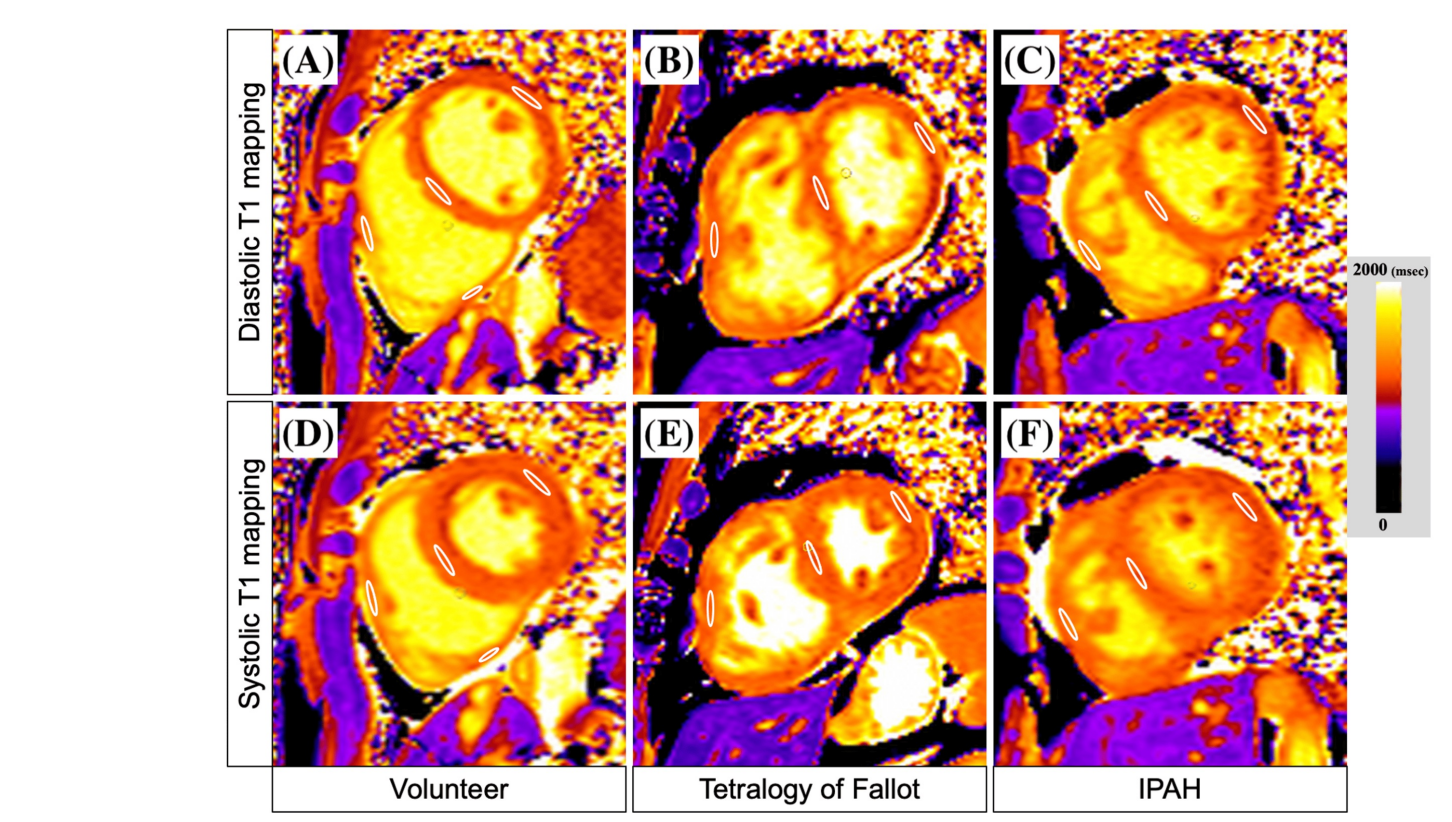
Diastolic and systolic T1 mapping images Diastolic T1 mapping: (A) volunteer, (B) tetralogy of Fallot, and (C) IPAH; systolic T1 mapping: (D) volunteer, (E) tetralogy of Fallot, and (F) IPAH Circles indicate measurement sites. iPAH, idiopathic pulmonary artery hypertension

**Figure 4:**
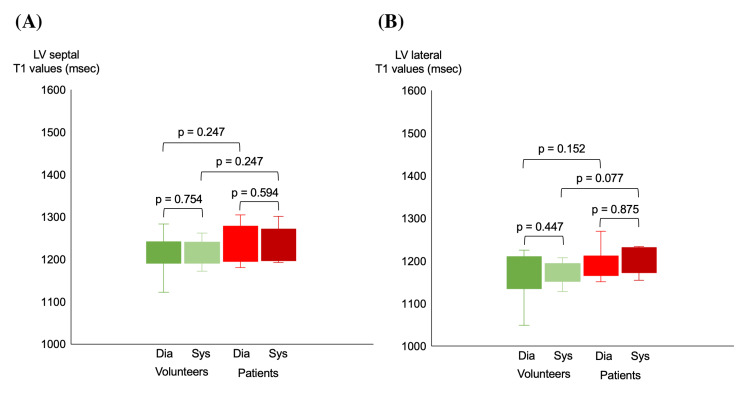
Comparison of left ventricular diastolic and systolic myocardial native T1 values Native T1 did not change significantly during the cardiac cycle in the (A) septal and (B) lateral walls of the left ventricle. No significant differences were found between the healthy and patient groups in the left ventricle. p < 0.05 was considered significant. Dia, diastolic phase; Sys, systolic phase

The characteristics of participants in this study are presented in Table 1. We investigated whether the nT1 changed because of the difference in the cardiac cycle using the left ventricular nT1 (Table 1, Figure 3, Figure 4).

**Table 1:** Demographic and T1 mapping data The values are presented as mean ± SD. * p < 0.05 was considered significant. Heart rate and systolic RV nT1 were significantly different between volunteers and patients (p = 0.03 and p = 0.002, respectively). LV, left ventricular; nT1, native T1 values; RV, right ventricular

Characteristic	Volunteers (n = 13)	Patients (n = 12)	p
Sex (male: female)	11: 2	8: 4	0.378
Age (years)	23.5 ± 1.3	21.7 ± 9.2	0.052
Height (cm)	167.2 ± 6.9	162.6 ± 11.3	0.347
Body weight (kg)	58.4 ± 6.9	52.9 ± 11.8	0.347
Heart rate (bpm)	70.7 ± 7.5	80.2 ± 11.5	0.030*
Diastolic			
LV septal nT1 (ms)	1,216.0 ± 40.5	1,238 ± 41.1	0.247
LV lateral nT1 (ms)	1,161.3 ± 49.9	1,120 ± 41.2	0.152
RV nT1 (ms)	1,347.6 ± 86.5	1,346.8 ± 96.7	0.852
Systolic			
LV septal nT1 (ms)	1,214.8 ± 27.1	1,238.2 ± 39	0.247
LV lateral nT1 (ms)	1,167.2 ± 37.1	1,199.2 ± 29.6	0.077
RV nT1 (ms)	1,233.8 ± 58.9	1,312.7 ± 34.5	0.002*

The diastolic nT1 of the ventricular septal wall in the 13 healthy subjects was 1,216.0 ± 40.5 ms, and the systolic nT1 was 1,214.8 ± 27.1 ms, showing no significant difference (p = 0.754) (Figure 4A). In addition, the diastolic nT1 of the lateral wall of the left ventricle was 1,161.3 ± 49.9 ms, and the systolic nT1 was 1,167.2 ± 37.1 ms, neither of which was significantly different (p = 0.447) (Figure 4B). We found that nT1 did not change significantly during the cardiac cycle. nT1 in the lateral wall of the left ventricle was significantly lower than that in the septal wall, both in the diastolic and systolic phases (diastole, p = 0.003; systole, p = 0.002). There was no difference in left ventricular nT1 by cardiac cycle in the patient group, nor was there a significant difference in left ventricular nT1 compared to the healthy subjects (Figure 4).

RV T1 mapping

**Figure 5:**
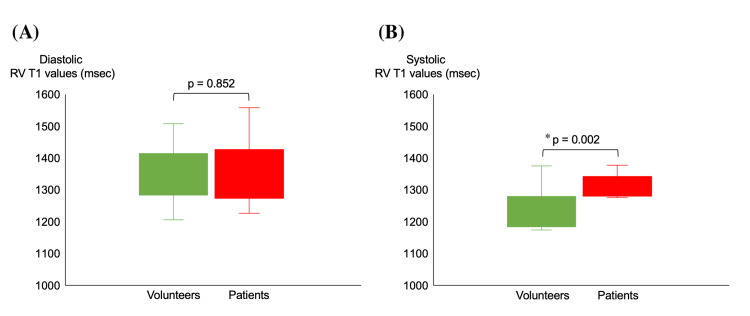
Comparison of right ventricular diastolic and systolic myocardial native T1 values (A) Diastolic RV T1 values were not significantly different between the healthy and patient groups, but (B) systolic RV T1 values were significantly higher in the patient group (p = 0.002). * p < 0.05 was considered significant. RV, right ventricular

Normal systolic right ventricular nT1 was measured using systolic right ventricular T1 mapping in the healthy subjects, and Figure 3 shows T1 mapping images of the healthy subject and patient group. The right ventricular nT1 was determined by setting ROIs in areas where the myocardium of the right ventricular free and inferior walls is sufficiently thick to avoid the effects of blood. As a result, the normal systolic right ventricular free and inferior walls nT1 were 1,234.8 ± 42.4 ms and 1,235.6 ± 36.7 ms, respectively (p = 0.795). Hence, normal right ventricular myocardium did not differ in systolic nT1 depending on the site of measurement. On the other hand, in the diastolic phase, the right ventricular nT1 in the healthy subject was 1,347.6 ± 86.5 ms, and the right ventricular nT1 in the right ventricular overloaded group was 1,346.8 ± 96.7 ms, which was no difference (p = 0.852, Figure 5A). In the systolic phase, the right ventricular myocardial nT1 in the healthy subject was 1,233.8 ± 58.9 ms, and the right ventricular nT1 in the right ventricular overloaded group was 1,312.7 ± 34.5 ms, which was a significant difference (p = 0.002, Figure 5B).

## Discussion

In this study, we found that systolic T1 mapping could be obtained from diastolic T1 mapping and cine MRI images; there was little change in nT1 due to the cardiac cycle, and the systolic right ventricular nT1 was high in patients with right ventricular overload.

Under standard settings, the cardiac cycle is determined from the QRS complex, and T1 mapping is performed during diastole [9,10]. Therefore, systolic imaging should be devised, as in this study. Our results suggest almost no difference in T1 mapping processing time between individuals, and systolic T1 mapping was possible by subtracting 185 ms from the systolic time of the cine MRI images. It is necessary to examine whether this value of 185 ms remains the same for other MRI devices in the future. Once the processing time for T1 mapping is obtained at one’s facility, systolic T1 mapping can always be obtained from cine MRI images, which is very useful. When setting the systolic time, Reiter et al. examined systolic T1 mapping by shifting a certain amount of time from diastole [11]. In that method, changes in heart rate cause a shift in systolic timing. Therefore, our method is better at setting systolic timing independent of heart rate.

In addition, we found that the nT1 hardly changed during the cardiac cycle. Tessa et al. reported no difference in left ventricular nT1 between diastole and systole [12]. In contrast, Reiter et al. reported that nT1 significantly decreased during systole than during diastole, but the difference was approximately 2.5%, which is unlikely to be clinically significant [11]. The slight change in nT1 with the cardiac cycle indicates no problem in evaluating the right ventricular nT1 in either the diastolic or systolic cardiac cycle. In determining the normal right ventricular nT1, it is difficult to measure the diastolic right ventricular nT1 in healthy subjects. Because the right ventricular myocardium has a thin myocardial wall and rough endocardial surface, the myocardium is insufficiently thick to surround the myocardial ROI and is susceptible to blood-induced T1 elevation. Because the myocardial wall is thicker during systole than diastole, the free and inferior walls of the right ventricle increase the number of sites where the ROI can be obtained. Despite this, measurement locations are often limited. When considering setting normal values, it is important to take multiple cross-sections to increase the number of measurable locations, as we did.

Regarding the site of myocardial T1 measurement, our results showed a significant difference between the left ventricular septal and posterior walls, consistent with a previous report [5]. nT1 of the left ventricular posterior wall is known to be lower than that of the septal wall owing to the effects of cardiac motion [5]. Similar results were expected for the right ventricular myocardium. However, there was no significant difference in nT1 between the right ventricular free and inferior walls during systole in healthy subjects. Therefore, if it is difficult to obtain the nT1 on the free wall, there is no significant problem in evaluating the nT1 on the inferior wall. It is desirable to evaluate the same location over time.

Consistent with previous reports, patients with right ventricular myocardial injury had elevated right ventricular nT1 [13,14]. The right ventricular nT1 is known to be elevated in patients with pulmonary hypertension and after surgery for tetralogy of Fallot. However, in previous reports, the right ventricular nT1 was evaluated during diastole [13,14]. The validity of the diastolic right ventricular nT1 has been cited as a limitation in all the previous reports. Among patients with right ventricular overload, especially those with right ventricular pressure overload, the thickness of the right ventricular myocardium is sufficient even during diastole due to right ventricular hypertrophy. Therefore, the measurement of nT1 is almost unaffected by blood and can be measured accurately. As mentioned earlier, it is challenging to measure the normal T1 value in the diastolic right ventricular myocardium. Without knowing normal values, it is not possible to evaluate right ventricular T1 values in disease groups. Even in patients with a thick right ventricular myocardium, it is possible to compare with normal values by imaging systolic T1 mapping, and quantitative evaluation of right ventricular myocardial damage can now be performed.

One of the limitations of this study is the small number of cases. In particular, there is limited data on healthy subjects. Since nT1 changes are known to depend on sex and age, it is necessary to increase the number of cases in the future and determine the normal value of right ventricular nT1 during systole. Also, due to the small number of cases, we were unable to separate patient groups into pressure or volume overloads. It is not known to what extent pressure or volume overload causes an increase in right ventricular nT1. It is necessary to study with a large number of patients in the future. Second, it is difficult to measure the right ventricular nT1. The right ventricular myocardium of healthy subjects was measured as a normal myocardium at any site. There might be a difference depending on the site of the patient group. To ensure sufficient thickness of the right ventricular myocardium, even during systole, it is considered appropriate to evaluate systolic T1 mapping only in patients with right ventricular overload, and further investigation is required.

## Conclusions

We quantitatively evaluated the properties of the right ventricular myocardium by right ventricular myocardial T1 mapping during systole. By considering the timing of imaging, systolic T1 mapping can be easily performed. Myocardial T1 values are indicators that reflect inflammation and fibrosis, and applying them to the right ventricle is an important technique that allows the evaluation of right ventricular myocardial damage. In the future, if changes in right ventricular nT1 are known due to pressure overload and volume overload in the right ventricle, early therapeutic intervention may be considered.
